# Real-Life Use of Component-Specific IgE in IgE-Mediated Cow’s Milk Protein Allergy in a Spanish Paediatric Allergy Centre

**DOI:** 10.3390/antib12040076

**Published:** 2023-11-17

**Authors:** Caoimhe Cronin, Cristina Muñoz Archidona, Beatriz Fernández Prudencio, Aoife Gallagher, Roberto Velasco Zuniga, Juan Trujillo Wurttele

**Affiliations:** 1 Department of Paediatrics and Child Health, University College Cork, T12 DC4A Cork, Ireland; 2 Irish Centre for Maternal and Child Health Research (INFANT), HRB Clinical Research Facility Cork (CRF-C), Cork University Hospital, T12 DC4A Cork, Ireland; 3 Hospital Universitario Severo Ochoa, 28914 Leganes, Spain; 4 Hospital Universitario de Móstoles, 28935 Madrid, Spain; 5 Department of Paediatrics, Cork University Hospital, T12 DC4A Cork, Ireland; 6 Paediatric Emergency Unit, Hospital Universitari Parc Tauli Barcelona, 08208 Barcelona, Spain

**Keywords:** food allergy, desensitisation, casein, alpha-lactalbumin, beta-lactoglobulin

## Abstract

Background: In Spain, IgE-mediated cow’s milk protein allergy (CMPA) affects approximately 0.69% of infants. Molecular diagnosis may be useful for monitoring natural spontaneous tolerance development in CMPA. The aim of this study was to retrospectively analyse a cohort of paediatric patients with IgE-mediated CMPA who were avoiding milk products awaiting natural tolerance and determine the relationship between disease persistence and major cow’s milk allergens. Methods: A retrospective chart review of 200 patients diagnosed with IgE-mediated CMPA between 2011 and 2020 was conducted. Patients strictly avoided milk products until an oral food challenge was performed. The main outcome was the introduction of liquid milk following a negative oral food challenge and its correlation with IgE and SPT measurements of milk components at diagnosis. Secondary outcomes included the rate of allergic reactions and anaphylaxis during the treatment period and its correlation with IgE and SPT measurements. Results: Of the 200 charts analysed, 122 patients had a negative oral food challenge to milk (61.0%) (95% confidence interval (CI): 54.1–67.5) following a period of strict avoidance of milk. Higher levels of component-specific IgE, especially casein, were associated with failure in the oral food challenge (*p* = 0.02). Allergic reactions were experienced by 106 children (53%), of which 34 (17%; 95% CI: 12.4–22.8) had anaphylactic reactions. The risk of anaphylaxis was not predicted by raised IgE levels. Conclusions: While a large proportion of children acquired natural tolerance to cow’s milk following a period of strict avoidance, IgE-mediated CMPA persisted in many children. Casein IgE levels at diagnosis were raised in those who failed to achieve natural tolerance. Allergic reactions to milk, including anaphylaxis, occurred commonly, but this was not predicted by raised IgE levels or SPT measurements.

## 1. Introduction

Cow’s milk protein allergy (CMPA) is one of the most common food allergies in infancy and childhood. Most cases spontaneously resolve over time, with 52.6% becoming tolerant to cow’s milk by 5 years [1]. In the EuroPrevall birth cohort study, the incidence of cow’s milk allergy in Spain was 0.69% [2]. CMPA is considered to have a high rate of resolution in childhood, with the resolution and acquisition of natural tolerance reported to be >50% by 5–10 years of age [3]. However, the disease duration varies between studies, suggesting that the natural history of CMPA is changing over time [3]. The increased persistence of CMPA has potential implications for its management and treatment [3].

Diagnosis of CMPA involves an assessment of the patient’s clinical history supported by routine testing involving skin prick tests (SPTs) and/or serologic testing of IgE antibodies to complete cow’s milk extract [4]. In some centres, the IgE antibodies to components of cow’s milk that are considered to be major allergens are also measured [4]. Cow’s milk contains caseins, beta-lactoglobulin, and alpha-lactalbumin, all of which are considered major allergens [5]. Molecular diagnosis may be useful for monitoring natural spontaneous tolerance development in CMPA [6], as it has been shown that lower serum levels of casein IgE are associated with higher chances of resolution of CMPA [7].

Whether IgE recognition profiles can have predictive value for disease progression in CMPA is an area being rigorously explored [5]. The aim of this study was to retrospectively analyse a cohort of paediatric patients with IgE-mediated CMPA who were avoiding milk products awaiting natural tolerance and determine the relationship between disease persistence and major cow’s milk allergens.

## 2. Methods

### 2.1. Study Population

Ethical approval was received from the Mostoles University Hospital Ethics Committee to conduct a retrospective chart review at Mostoles University Hospital Madrid, Spain.

Paediatric patients diagnosed with IgE-mediated CMPA between 2011 and 2020 were identified. IgE-mediated symptomatology was identified by cutaneous (urticaria and angioedema, and eye irritation), gastrointestinal (vomiting, abdominal pain, and diarrhoea), and/or respiratory (rhinorrhoea, nose itchiness, cough, wheezing, sore throat, and respiratory distress) symptoms.

The inclusion criteria for this study were children with a diagnosis of IgE-mediated CMPA who met the diagnostic criteria of the presence of cutaneous, gastrointestinal, respiratory, and/or multisystem (anaphylaxis) symptoms consistent with IgE-mediated CMPA after the ingestion of formula milk and one of the following:

- Positive skin prick test for cow’s milk (≥3 mm);
- Specific IgE levels for cow’s milk and or milk components of >0.35 k U/L.

Cases were excluded if the data were considered incomplete. Any child who had already tolerated baked milk was included.

### 2.2. Intervention

Patients with a diagnosis of IgE-mediated CMPA were immediately entered into a strict avoidance of milk and milk products. Breastmilk was encouraged with or without combination with extensively hydrolysed milk or amino-acid-based milk formulas. However, if a patient had any direct reaction to breastmilk, avoidance of breastfeeding was also necessary. Follow-up appointments were scheduled every 6–12 months and included clinical appointments and allergy tests, such as skin prick tests and, if needed, oral food challenges, according to the allergy consultant’s indication. The decision on the duration of avoidance of milk was made after receiving clinical and allergy test results. A 50% or more drop in cow’s-milk-specific IgE levels over 1–2 years was considered a favourable prognostic indicator of natural tolerance.

### 2.3. Outcome Measurements

The primary outcome was the introduction of unrestricted liquid cow’s milk after passing an oral food challenge and the association with milk component SPT and IgE levels at diagnosis. Secondary outcomes included experiencing allergic reactions due to accidental exposure to milk and anaphylaxis as a result of accidental exposure to milk.

### 2.4. Clinical Characterisation of Subjects

Demographic data (sex, mode of delivery, breastfeeding history, and family history of allergic conditions) were collected for analysis. Clinical details included skin prick test wheal size and milk-component-specific IgE levels at diagnosis, age at the start and finish of reintroduction, the number of clinic appointments, and history of other atopic conditions (other food allergies, atopic dermatitis, allergic rhinitis, and asthma/viral-induced wheeze). IgE measurements were taken using blood samples and analysed using the ImmunoCAP^®^ Phadia^®^ Laboratory System for whole IgE and component-specific IgE levels for alpha-lactalbumin, beta-lactoglobulin, and whole casein.

### 2.5. Data Analysis

Descriptive statistical analysis was carried out for all collected variables. Continuous data were expressed as means with a 95% confidence interval (CI), whereas categorical variables were reported as percentages. Comparisons of the qualitative data between patients with or without symptoms of anaphylaxis at diagnosis were carried out using chi-square. Data were considered statistically significant if the *p*-value was ≤0.05. Univariate analysis using chi-square and one-way ANOVA and multivariate analysis using binary logistic regression were used to assess the successful acquisition of natural tolerance with milk component measurements and other clinical factors, including a history of other food allergies and carrying an adrenaline autoinjector (AAI). Odds ratios (ORs) were expressed with 95% CIs. Variables associated with an outcome in the univariate analysis (*p*-value < 0.05) were considered for the multivariate model, and the final model was selected using stepwise regression (*p*-value < 0.05). To compare specific IgE levels at diagnosis and following the OFC, the means were compared using a genialised linear model. Data were analysed with SPSS Version 28.

## 3. Results

### 3.1. Study Population

The total cohort of patients in the 10-year analysis included 200 patients with IgE-mediated CMPA.

The baseline demographic features are shown in Table 1. A majority of 132 (66%) had a significant family history of atopy. As well as a diagnosis of CMPA, 122 (61%) had atopic dermatitis, 95 (47.5%) had viral-induced wheeze, 39 (19.2%) had allergic rhinitis, and 79 (39.5%) had other food allergies. Food allergies included egg (63 (31.5%)), peanut (13 (6.5%)), other nuts (13 (6.5%)), and other food (26 (13%)) allergies. Of the 200 patients enrolled, 95 (47.5%) had been prescribed adrenaline autoinjectors.

### 3.2. Primary Outcome

#### 3.2.1. Completion of Treatment

Of the 200 patients in the cohort who were advised to strictly avoid cow’s milk followed by an oral food challenge, 122 (61.0%; 95% CI: 54.1–67.5) passed the oral food challenge and successfully introduced cow’s milk into their diet. The median time of clinically recommended cow’s milk avoidance was 21 months (IQR 12–37), during which time, patients attended a median of 5 (IQR 4–6) appointments. The median age at which tolerance was achieved was 26 months (IQR 18–42 months).

Of the 200 patients enrolled, 52 patients (26%) acquired natural tolerance by 2 years of age, 87 children (44%) achieved tolerance by 3 years of age, and 100 children (50%) had achieved tolerance by 4 years of age. By the school-starting age of 5 years, 114 children (57%) had achieved natural tolerance to cow’s milk.

The univariate analysis of demographic factors associated with success in achieving natural tolerance found statistically significant associations with a diagnosis of asthma/viral-induced wheeze, allergic rhinitis, egg allergy and peanut allergy, carrying an adrenaline autoinjector, experiencing allergic reactions during treatment, and experiencing anaphylaxis during treatment (Table 2). In the multivariate analysis, it was found that carrying an AAI, experiencing allergic symptoms upon accidental exposure to milk, and being treated for anaphylaxis were associated with a reduced likelihood of acquiring natural tolerance to cow’s milk (OR 0.148, 0.396, and 0.066, respectively).

#### 3.2.2. Skin Prick Test and Milk-Component-Specific IgE Levels

Skin prick tests and serum-specific IgE measurements were performed in almost all patients at diagnosis (*n* = 194 and *n* = 200, respectively). In the univariate analysis, increased skin prick test wheal diameters and raised specific IgE levels were associated with an increased risk of failure to achieve natural tolerance (Table 3). Multivariate analysis was performed with anaphylaxis during treatment included as a confounding factor. This multivariate analysis using binary logistic regression demonstrated that raised casein-specific IgE levels increased the risk of failure to achieve natural tolerance during the study period (OR 1.129 (95% CI: 1.019–1.251); *p* = 0.02) (Table 3).

**Figure 1:**
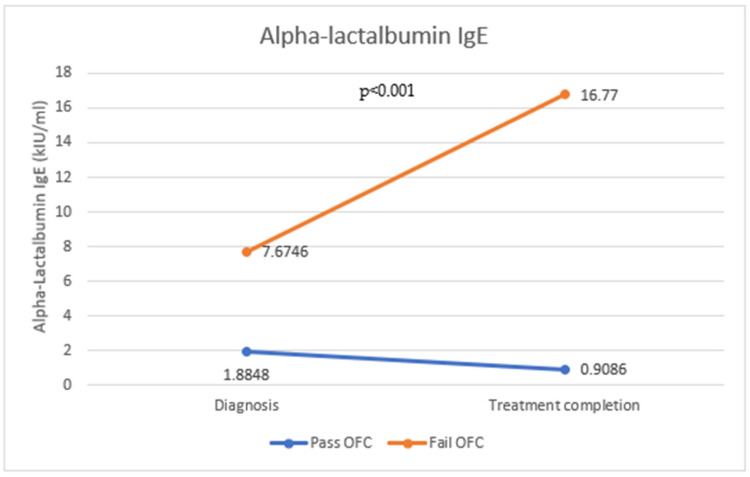
Change in alpha-lactalbumin IgE in those who passed and failed the OFC.

**Figure 2:**
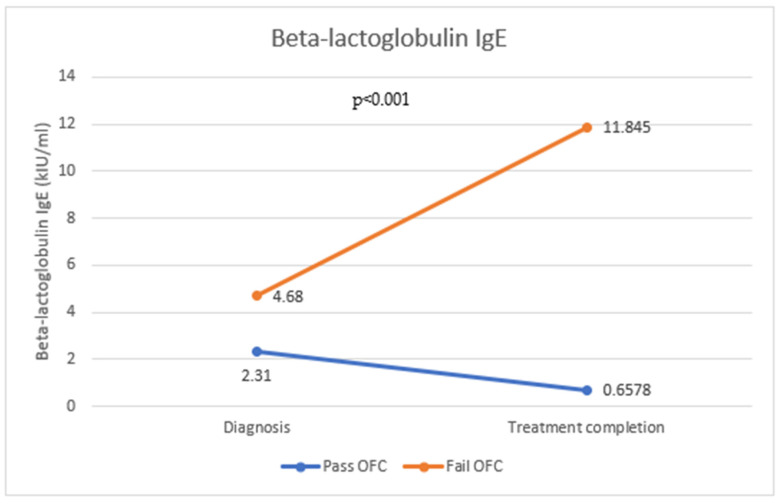
Change in beta-lactoglobulin IgE in those who passed and failed the OFC.

**Figure 3:**
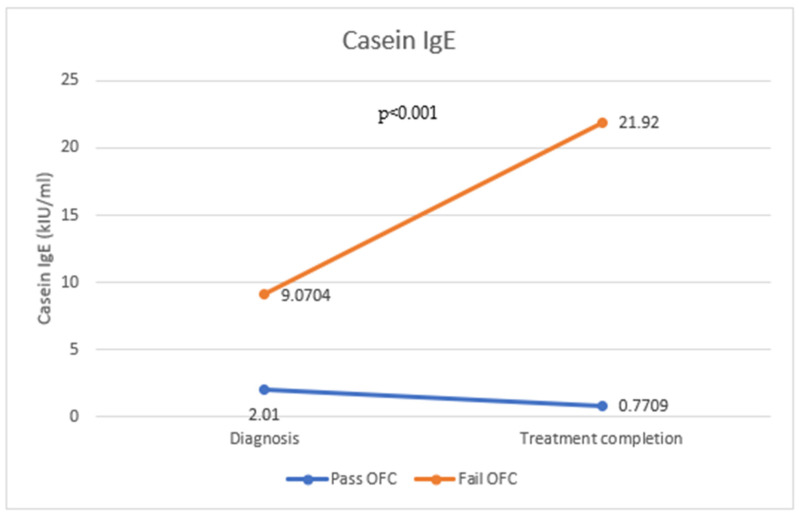
Change in casein IgE levels in those who passed and failed the OFC.

Specific IgE levels were measured for alpha-lactalbumin (Figure 1), beta-lactoglobulin (Figure 2), and casein (Figure 3) in 173 patients following the oral food challenge conducted following the period of strict milk avoidance. There was a statistically significant change in all IgE components in those who passed or failed the OFC. For all three milk components, IgE levels were reduced in those who passed the OFC, while those who failed the OFC showed significantly raised IgE levels compared with levels at diagnosis (*p* < 0.001).

### 3.3. Secondary Outcomes

#### Safety

The number of patients who experienced allergic reactions to cow’s milk during the period of milk avoidance was 106 (53.0%; 95% CI: 46.1–59.8). Of these, 34 patients (17.0%; 95% CI: 12.4–22.8) were treated for anaphylaxis. The most common symptom manifested during these allergic reactions was urticaria (*n* = 72), followed by vomiting (*n* = 36) and wheezing (*n* = 24). Other symptoms experienced included angioedema (*n* = 21), stridor (*n* = 15), rhinitis (*n* = 13), and abdominal pain (*n* = 9).

In the univariate analysis, children with a diagnosis of asthma/viral-induced wheeze were more likely to experience allergic reactions (85.2% vs. 14.7%; OR 8.788 (95% CI: 3.237–23.85); *p* < 0.001). Children with a concomitant diagnosis of allergic rhinitis (35.3% vs. 16.3%; OR 2.808 (95% CI: 1.243–6.346); *p* < 0.011) and egg allergy (47.1% vs. 28.3%; OR 2.251 (95% CI: 1.06–4.78); *p* = 0.032) were also more likely to experience anaphylaxis. Similarly, children who carried an adrenaline autoinjector were more likely to experience anaphylaxis during treatment (82.4% vs. 25.9%; OR 13.349 (95% CI: 5.175–34.435); *p* < 0.001). There were no such associations between gender, prematurity, atopic dermatitis, peanut allergy, or experiencing anaphylaxis prior to diagnosis.

The univariate analysis of skin prick test wheal diameter and serum-specific IgE levels at diagnosis found that SPTs for whole milk and casein and casein-specific IgE levels were raised in those who were treated for anaphylaxis during treatment (Table 4). The multivariate analysis, which included the demographic factors above that had a statistically significant association with anaphylaxis during treatment, revealed that only a diagnosis of asthma/viral-induced wheeze (OR 9.068 (95% CI: 2.69–30.58); *p* < 0.001) and carrying an AAI (OR 8.136 (95% CI: 2.606–25.403); *p* < 0.001) were associated with an increased risk of experiencing anaphylaxis while strictly avoiding cow’s milk.

## 4. Discussion

In this study, we retrospectively reviewed patients with an IgE-mediated food allergy who were advised to strictly avoid milk and subsequently underwent an oral food challenge to assess for natural tolerance acquisition over a ten-year period in 2011–2021. The results show that 61% of patients acquired natural tolerance by a median age of 26 months. It was also found that component-specific IgE levels, particularly casein IgE, were effective in identifying children less likely to acquire natural tolerance during the study period. It was also discovered that 17% of patients experienced anaphylaxis during the treatment period.

The proportion of patients who achieved natural tolerance to milk followed the recognised pathway of tolerance acquisition in infants with CMPA [3], with 26% of children achieving tolerance by 2 years of age, 44% of children achieving tolerance by 3 years of age, and 57% of the total cohort achieving tolerance by the school-starting age of 60 months. While IgE-mediated CMPA was once considered a transient food allergy in infancy [8], these findings support the results of other studies demonstrating that natural tolerance acquisition is not heterogeneous, with tolerance acquisition at two years ranging from 20 to 77% and at five years ranging from 52 to 92% [8]. Thus, the continued persistence of CMPA, as shown in this study, into later childhood should be taken into consideration when discussing management.

This study addressed the employment of the measure of cow’s milk components in skin prick testing and serum IgE in predicting failure to acquire natural tolerance. While all milk component parameters tested were raised in those who failed to achieve natural tolerance during the study period, multivariate analysis revealed that increased serum casein IgE at diagnosis was the main component that predicted failure to achieve natural tolerance. In addition, serum casein IgE was raised in those who failed the oral food challenge. These findings are similar to those in a prospective study in which CMPA diagnosis was confirmed with DBPCFC to cow’s milk, where children with lower serum levels of IgE specific to CM, alpha-lactalbumin, beta-lactoglobulin, kappa-casein, and alpha s1-casein had a greater likelihood of outgrowing CMPA over an 80-month period [9]. However, children with persistent milk allergy have been shown to predominantly generate IgE antibodies directed against casein epitopes [6,10,11]. The similarly raised casein IgE levels in those who failed the OFC followed the pattern of stable IgE epitope-binding patterns in patients with persisting CMPA, while binding decreased in patients who recovered early [4]. Therefore, while EAACI does not recommend the routine use of molecular diagnostics for standard evaluation of suspected CMPA [5], the measurement of serum casein IgE may identify children at risk of delayed tolerance acquisition who may benefit from further intervention to reduce the risk of severe allergic reactions in later life.

While complete avoidance of milk has been the most widespread management strategy for CMPA, the European Academy of Allergy and Clinical Immunology (EAACI), recommends oral immunotherapy (OIT) for children aged 4–5 years with persistent CMPA [12]. In recent years, the safety and effectiveness of OIT in infants of less than 12 months with IgE-mediated CMPA have been explored in several pilot studies, with satisfactory safety and effectiveness values ranging from 87.5 to 100% [8]. By identifying infants at high risk of CMPA persistence at diagnosis, such as those with raised casein IgE levels, or those at higher risk of experiencing anaphylaxis, such as those with asthma/viral-induced wheeze, the early introduction of milk using OIT promotes the acquisition of tolerance in those who may have had an allergy persisting into later life.

Additionally, dietary advancement therapies, including the milk ladder, have been shown to be successful in several studies, with 90% of patients in a retrospective study in the UK achieving the top step of the ladder and achieving tolerance [13], and over 83% of patients successfully introducing milk into their diet in an analysis of the employment of the milk ladder in a tertiary allergy centre and a primary care setting in Ireland [14].

The proposed mechanism of the milk ladder is the process of baking milk, which alters the structure of milk allergens, changing their stability and subsequently creating decreased allergenicity (via decreased IgE binding) [8]. Regular consumption of these baked milk products therefore decreases IgE levels, allowing for increased tolerance to milk proteins as the degree of allergenicity increases through the milk ladder [8]. This mechanism of action is supported by the findings of this study, where those who passed the OFC experienced a reduction in specific IgE levels over time. Caseins, which are composed of four major proteins, α_s1_-casein (Bos d 9), α_s2_-casein (Bos d 10), β-casein (Bos d 11), and κ-casein (Bos d 12), that exist in a colloidal particle known as the casein micelle [15,16], are more resistant to heating compared with whey proteins, which are susceptible to heating. This is attributed to the linkage between phosphoserine-containing caseins formed by calcium bridges and linkages with colloidal calcium phosphate within the casein micelles [15]. Therefore, high levels of specific IgE antibodies directed against casein are predictive of clinical reactivity to baked milk. Therefore, the identification of raised casein IgE levels, indicating children who are reactive to baked milk, may identify children who may benefit from the gradual introduction of baked milk using the milk ladder or similar dietary advancement therapy.

An analysis of the safety of the dietary avoidance of cow’s milk demonstrated that over half of patients experienced allergic reactions to milk, with 17% of patients being treated for anaphylaxis during this period. The rate of anaphylaxis in this cohort who were advised to avoid milk was higher than in those undergoing oral immunotherapy and dietary advancement therapies via the milk ladder [8,12]. The burden of morbidity and mortality from anaphylaxis to food in this age cohort cannot be underestimated [17], and adequate caregiver education and dietetics input are invaluable when advising patients and their families on avoiding milk [18].

In the binary regression analysis, the concomitant diagnosis of asthma/viral-induced wheeze and the carrying of an adrenaline autoinjector increased the risk of being treated for anaphylaxis during the period of avoidance, with asthma being a recognised risk factor for severe allergic reactions causing anaphylaxis [19]. The carrying of an AAI increasing the risk of being treated for anaphylaxis is speculated to be a confounding factor. Raised levels of milk components did not predict the risk of anaphylaxis during treatment.

The strengths of this study include the large sample size of 200 patients, increasing the external validity of this study and creating a cohort that is representative of the broader population. The broad study period of 2011–2021 for the retrospective clinical data provides an insightful analysis of the real-life application of measuring the components of cow’s milk and the impact of the management strategy on patients’ outcomes and quality of life by documenting allergic reactions.

There are several limitations to this study. Firstly, as this was a retrospective chart review, a prospective cohort would provide a more accurate representation of the treatment of CMPA from diagnosis to treatment outcome and follow-up. Secondly, this was a single-centre study; therefore, the applicability of the findings of this study in other paediatric centres may be limited.

## 5. Conclusions

In conclusion, this retrospective study demonstrated that while a large proportion of children acquired natural tolerance to cow’s milk following a period of strict avoidance, IgE-mediated CMPA persisted in many children. Casein IgE levels at diagnosis were raised in those who failed to achieve natural tolerance and failed the oral food challenge. Allergic reactions to milk, including anaphylaxis, commonly occurred, but this was not predicted by raised IgE levels or SPT measurements. This study contributes to the current literature on employing molecular diagnostics in the management of CMPA in a real-life paediatric cohort. Prospective cohort studies are recommended to further establish the role of specific IgE, especially casein IgE, in identifying children who may benefit from alternative management strategies for CMPA, such as OIT or dietary advancement therapies.

## Figures and Tables

**Table 1:** Patient demographics.

	Patients *(n* = 200)
Sex—male	126 (63%)
Age in months at diagnosis—**median (IQR)**	5 (4–7)
Prematurity	4 (2%)
Feeding before diagnosis	
Breast fed	181 (90.5%)
Bottle fed	10 (5%)
Mixed	9 (4.5%)
Unknown	0 (0%)
Duration of breastfeeding (if breastfed) in months	5 (4–6)
Atopic dermatitis	122 (61%)
Asthma/viral-induced wheeze	95 (47.5%)
Allergic rhinitis	39 (19.2%)
Any food allergy	79 (39.5%)
Egg	63 (31.5%)
Peanut	13 (6.5%)
Other nuts	13 (6.5%)
Other food	26 (13%)
Any family history of atopic conditions	132 (66%)
Food allergy	36 (18%)
Atopic dermatitis	32 (16%)
Asthma	52 (26%)
Allergic rhinitis	82 (41%)
Carry autoinjectors	95 (47.5%)

**Table 2:** Univariate and multivariate analysis of patient and treatment factors associated with successful acquisition of natural tolerance. * Factors with statistical significance (*p* < 0.05) that were included in the multivariate analysis. ^¥^ Factors that were found to be statistically significant (*p* < 0.05) in multivariate analysis.

	Univariate Analysis		Multivariate Analysis	
	Success *n* = 122 (%)	*p*-Value	OR (95% CI)	*p*-Value
Sex		0.390		
Male	74 (58.7)			
Female	48 (64.9)			
Prematurity		0.106		
Yes	4 (100)			
No	118 (60.2)			
Atopic dermatitis		0.107		
Yes	69 (56.6)			
No	53 (67.9)			
Asthma/viral-induced wheeze		<0.001 * OR 0.248 (95% CI: 0.135–0.453)	OR 0.583 (0.259–1.315)	*p* = 0.194
Yes	42 (44.2)			
No	80 (76.2)			
Allergic rhinitis		<0.001 * OR 0.205 (95% CI: 0.097–0.439)	OR 0.612 (0.218–1.722)	*p* = 0.353
Yes	12 (30.8)			
No	110 (68.3)			
Egg allergy		0.003 * OR 0.404 (95% CI: 0.219–0.744)	OR 0.856 (0.354–2.069)	*p* = 0.730
Yes	29 (46)			
No	93 (67.9)			
Peanut allergy		0.004 * OR 0.171 (95% CI: 0.046–0.644)	OR 0.492 (0.1–2.427)	*p* = 0.384
Yes	3 (23.1)			
No	119 (63.6)			
Anaphylaxis prior to diagnosis		0.732		
Yes	5 (55.6)			
No	117 (61.3)			
Carry autoinjector		<0.001 * OR 0.063 (95% CI: 0.031–0.129)	OR 0.148 (0.06–0.367)	*p* < 0.001 ^¥^
Yes	16 (22.5)			
No	106 (82.2)			
Allergic reaction to milk during treatment (accidental exposure)		<0.001 * OR 0.224 (95% CI: 0.12–0.418)	OR 0.396 (0.178–0.881)	*p* = 0.023 ^¥^
Yes	48 (45.3)			
No	74 (78.7)			
Anaphylaxis during treatment (accidental exposure)		<0.001 * OR 0.024 (95% CI: 0.006–0.104)	OR 0.066 (0.013–0.331)	*p* < 0.001 ^¥^
Yes	2 (5.9)			
No	120 (72.3)			

**Table 3:** Univariate and multivariate analysis of SPT (mm) and specific IgE levels (kIU/mL) with successful acquisition of natural tolerance. * Factors with statistical significance (*p* < 0.05) that were included in the multivariate analysis. ^¥^ Factors that were found to be statistically significant (*p* < 0.05) in multivariate analysis.

	Univariate Analysis			Multivariate Analysis	
	Success (Mean (95% CI))	Fail (Mean (95% CI))	*p*-Value	OR (95% CI)	*p*-Value
Skin prick tests (SPTs) at diagnosis					
SPT—whole milk (mm)	2.43 (2.01–2.84)	3.63 (2.90–4.36)	0.002 *	1.036 (0.848–1.265)	0.732
SPT—alpha-lactalbumin (mm)	3.17 (2.566–3.78)	4.33 (3.44–5.21)	0.028 *	1.017 (0.891–1.16)	0.804
SPT—beta-lactoglobulin (mm)	3.33 (2.82–3.85)	4.3 (3.39–5.21)	0.05 *	1.047 (0.905–1.211)	0.537
SPT—casein (mm)	2.16 (1.75–2.58)	3.33 (2.64–4.01)	0.002 *	0.984 (0.789–1.227)	0.884
Specific IgE levels at diagnosis					
Alpha-lactalbumin-specific IgE (kIU/mL)	1.844 (1.05–2.62)	7.59 (4.65–10.53)	<0.001 *	1.08 (0.98–1.189)	0.12
Beta-lactoglobulin-specific IgE (kIU/mL)	2.29 (1.52–3.06)	5.31 (3.16–7.46)	0.003 *	1.014 (0.937–1.096)	0.734
Casein-specific IgE (kIU/mL)	1.93 (1.28–2.59)	9.45 (6.43–12.47)	<0.001 *	1.129 (1.019–1.251)	0.02 ^¥^

**Table 4:** Univariate and multivariate analysis of SPT (mm) and specific IgE levels (kIU/mL) associated with treatment for anaphylaxis during period of milk avoidance. * Factors with statistical significance (*p* < 0.05) that were included in the multivariate analysis.

	Univariate Analysis			Multivariate Analysis	
	Anaphylaxis (Mean (95% CI))	No Anaphylaxis (Mean (95% CI))	*p*-Value	OR (95% CI)	*p*-Value
Skin prick tests (SPTs) at diagnosis					
SPT—whole milk (mm)	3.84 (2.64–5.04)	2.7 (2.31–3.1)	0.029 *	0.977 (0.782–1.220)	0.836
SPT—alpha-lactalbumin (mm)	4.21 (2.83–5.6)	3.5 (2.9–4.05)	0.306		
SPT—beta-lactoglobulin (mm)	4.12 (2.85–5.39)	3.63 (3.11–4.14)	0.449		
SPT—casein (mm)	3.5 (2.45–4.54)	2.44 (2.05–2.83)	0.037 *	0.938 (0.738–1.194)	0.605
Specific IgE levels at diagnosis					
Alpha-lactalbumin-specific IgE (kIU/mL)	6.14 (1.86–10.42)	3.66 (2.36–4.96)	0.155		
Beta-lactoglobulin-specific IgE (kIU/mL)	4.5 (1.58–7.43)	3.26 (2.24–4.28)	0.344		
Casein-specific IgE (kIU/mL)	9.17 (5.17–13.16)	3.99 (2.62–5.36)	0.004 *	0.98 (0.938–1.024)	0.361

## Data Availability

The data presented in this study are available only upon request from the corresponding author due to restrictions, i.e., privacy and ethics.

## Abbreviations

CMPA	Cow’s milk protein allergy
OFC	Oral food challenge
SPT	Skin prick test
CI	Confidence interval
AAI	Adrenaline autoinjector
EAACI	European Academy of Allergy and Clinical Immunology
OIT	Oral immunotherapy
